# *In vitro* efficacy of gentamicin released from collagen sponge in eradication of bacterial biofilm preformed on hydroxyapatite surface

**DOI:** 10.1371/journal.pone.0217769

**Published:** 2019-06-04

**Authors:** Beata Maczynska, Anna Secewicz, Danuta Smutnicka, Patrycja Szymczyk, Ruth Dudek-Wicher, Adam Junka, Marzenna Bartoszewicz

**Affiliations:** 1 Department of Pharmaceutical Microbiology and Parasitology, Wroclaw Medical University, Wrocław, Poland; 2 Centre for Advanced Manufacturing Technologies (CAMT/FPC), Faculty of Mechanical Engineering, Wrocław University of Science and Technology, Wrocław, Poland; University of Texas at Austin, UNITED STATES

## Abstract

Biofilm-related infections of bones pose a significant therapeutic issue. In this article we present *in vitro* results of the efficacy of gentamicin released from a collagen sponge carrier against *Staphylococcus aureus*, *Pseudomonas aeruginosa* and *Klebsiella pneumoniae* biofilms preformed on hydroxyapatite surface. The results indicate that high local concentrations of gentamicin released from a sponge eradicate the biofilm formed not only by gentamicin-sensitive strains but, to some extent, also by those that display a resistance pattern in routine diagnostics. The data presented in this paper is of high clinical translational value and may find application in the treatment of bone infections.

## Introduction

It is believed that 60–80% of nosocomial infections are caused by biofilm pathogens. Therefore, detection and treatment of pathogenic biofilm is among the most significant healthcare issues [1]. The extracellular matrix of biofilm contributes to its high tolerance to the host’s defense mechanisms, antibiotics and antiseptics [2,3]. Chronic infections of wounds and bones are also caused by biofilms [4–6]. The Gram-positive coccus, referred to as the *Staphylococcus aureus*, is considered the most ubiquitous etiological factor of such infections regardless its origin (nosocomial or community-acquired type). In turn, Gram-negative bacteria (*Pseudomonas aeruginosa* and *Enterobacteriacae* family members) occur more frequently in hospital-acquired infections [7–9]. Treatment of bone infections is a significant diagnostic and therapeutic problem. The specific anatomical structure of bone considerably limits the efficacy of antimicrobial measures and hinders the immune response [10–12]. Also, microbiological examination of bones is difficult due to problems with obtaining the appropriate diagnostic material. As regards treatment procedures, antibiotic therapy may raise some objections mostly because high doses of active agents (above the Minimum Inhibitory Concentration, MIC), required to be delivered to the infection site, may cause systemic toxicity [13]. Implants saturated with gentamicin represent an important exception to the above rule. Numerous data indicate that the high concentration of gentamicin released locally from the implant does not contribute to the high systemic concentration of this antibiotic [12,14,15]. Gentamicin belongs to a class of antibiotics referred to as aminoglycosides, which are still commonly used to treat severe infections, especially in combination therapy. According to EARSS (European Antimicrobial Resistance Surveillance System) data from 2015 [16], resistance against aminoglycosides among Gram-negative rods *P*.*aeruginosa* and *E*.*coli* is still relatively low (30% and 11%, respectively). However, an upward trend is currently observed. Other Gram-negative rods, such as *K*.*pneumoniae* and *Acinetobacter baumannii*, display higher resistance frequencies (59% and 70%, respectively). Aminoglycosides have such functional advantages as rapid bactericidal effect [1-2h], post-antibiotic effect [PAE], inoculum-independent activity, synergy with beta-lactam and glycopeptide antibiotics as well as easy dosing (one dose/day) [17]. On the other hand, there exist numerous microbial resistance patterns to aminoglycosides including enzymatic, receptor and transport mechanisms [17,18,19, 20]. Moreover, a systemic application of gentamicin in bone infection treatment is limited due to a low penetration ratio. Therefore, a number of approaches has been developed to increase this functional parameter. The most prominent of them include introduction of gentamicin with such natural carriers as albumins, collagens, chitosans, hyaluronic acids or with such synthetic carriers as polylactic acids, glycols, phosphates and hydroxyethylocellulose. All these approaches are designed to increase the antibiotic penetration through the biofilm and to allow a gradual release of the antimicrobial [21]. Clinical data suggest that the following are the indications for the application of gentamicin sponge: osteomyelitis and other bone infections, prophylaxis during procedures at risk of infection (implantations, bone grafts, surgical procedures at infection sites), proctologic surgery and cardiac surgery including bridge infections [22]. Purified I and III type collagen (from bovine tendons) is used in the gentamicin sponge [23, 24]. This natural polymer displays both low allergenicity and is biodegradable. Thus, as a carrier for gentamicin, a collagen sponge may be considered a fully biocompatible product. The biodegradation of the carrier eliminates the need of another surgery, accelerates wound healing and provides gradual and systematic gentamicin release [12,15]. It was previously demonstrated that gentamicin is released completely from the carrier during the first 60 min after implantation [12]. The obtained concentrations exceeded the established MIC and reached the value of 1000mg/L. During the next 4–5 days after implantation, the antibiotic concentration was at the level of 300-400mg/L [25]. However, in the serum, the measured gentamicin concentration was very low (below or equal to 2mg/L), which reduces the risk of systemic adverse effects, such as neuro- or nephrotoxicity) [12,15, 26]. Very high local concentration of the antibiotic suggests that also microorganisms of reduced sensitivity to gentamicin could be eradicated [18,19,20]. Therefore, the aim of this research was to evaluate the *in vitro* efficacy of high doses of gentamicin delivered locally via collagen sponge against bone pathogens.

## Materials and Methods

### Strains

45 bacterial strains from Strains’ Collection of Pharmaceutical Microbiology and Parasitology Department of Medical University of Wroclaw were used in this study. These strains were Gram-positive cocci: *S*.*aureus* (n = 14, isolated from sternal osteomyelitis and diabetic foot infections); and Gram-negative rods: *P*.*aeruginosa* (n = 14, isolated from sternal osteomyelitis and chronic leg ulcers) and *K*.*pneumoniae* (n = 17, isolated from deep chronic leg ulcers). The above-mentioned microorganisms were isolated from bone and wound infections from patients hospitalized in Wroclaw, Kolobrzeg and Krapkowice in the years 1994–2015.

Gentamicin used for experiments: the following forms of antibiotic were used in the experiments:

1. gentamicin sulfate (Sigma Aldrich, Germany) consisting of 660μg of antibiotic/1000mg of product)
2. E-test strips (bioMerieux, Poland) with antibiotic concentration gradient of 0.016-256mg/L
3. the 10x10x0.5 cm Garamycin Sponge (EUSA Pharma, Poland) consisting of 1.3mg/cm^2^ of purified type I and III (95%, 5%, respectively) collagen isolated from bovine tendons and saturated with 2.8mg/cm^2^ of gentamicin sulphate.

### Hydroxyapatite discs

Commercially available HA powder was used for custom disc manufacturing. Powder pellets of 9.6mm diameter were pressed without a binder. Sintering was performed at 900°C. The resulting tablets were compressed using the Universal Testing System for static tensile, compression, and bending tests (Instron model 3384; Instron, Norwood, MA). The quality of the manufactured HA discs was checked by confocal microscopy and micro–computed tomography (microCT) using a LEXT OLS4000 microscope (Olympus, Center Valley, PA) and Metrotom 1500 microtomograph (Carl Zeiss, Oberkochen, Germany).

### Antibiotic sensitivity testing

Kirky-Bauer method (standard disc diffusion testing) was performed to estimate the sensitivity of the analyzed microbes towards clinically used antibiotics according to EUCAST binding guidelines (www.eucast.org).

### E-test method

The Minimal Inhibitory Concentration of gentamicin against the tested microorganisms was analyzed using routine E-test method. Strips (bioMerieux, Poland) saturated with antibiotic gradient were placed on a Mueller-Hinton agar plate containing a pathogen culture. The inhibition of microbial growth at specific antibiotic concentration was assessed according to binding EUCAST guidelines. Resistance [R] and sensitivity [S] were established using the following breakpoints for *P*.*aeruginosa*: S≤4mg/L, R>4mg/L, for *S*.*aureus* S≤1mg/L, R>1mg/L and for *K*.*pneumoniae* S≤2mg/L, I: 2-4mg/L, R>4).

### Serial microdilution method

This technique, performed in a 96-well plate, was applied to compare the sensitivity of planktonic (Minimal Inhibitory Concentration, MIC) and biofilm forms of bacteria (Minimal Biofilm Eradication Concentration, MBEC) to gentamicin. Briefly, in the case of planktonic assessment, the strains were cultured into an appropriate liquid medium and incubated at 37°C for 24 hours. Next, optical density (expressed in McFarland scale) was measured using a densitometer (Biomerieux, Poland). The culture was diluted in the medium to 1×10^5^ cells/ml). Subsequently, the antibiotic solutions in concentration of 500mg/L -1mg/L were transferred to the adjacent wells of a 96-well polystyrene plate. Next, 100μL of bacterial suspension was added to each well. The plate was incubated for 24h/37°C. Afterwards, 2μL of 1% triphenyltetrazolium chloride (TTC, Sigma-Aldrich) was added to the wells. Reduction of colorless TTC to red formazan confirmed the presence of metabolically active microorganisms in the plate’s well. The first colorless well of the plate showed antibiotic MIC. As regards biofilm measurement, the experiment was performed analogically, the difference being that a strain culture was first allowed to form biofilm on the bottom of a well’s plate for 24 hours and then the medium containing various concentrations of the antibiotic was added. All of the procedures were performed in triplicates.

### Confirmation of biofilm formation on HA disc by Scanning Electron Microscopy

Sterile HA discs were placed into the wells of a 24-well plate. Next, 2mL of 1×10^5^ cells/ml of a particular pathogen was introduced into this setting and left for 24h/37°C. After incubation, the surface of the HA discs was gently rinsed using physiological saline solution to remove non-adherent organisms and to leave the biofilm structure only. Subsequently, the discs were fixed using 3% glutarate (Poch, Poland) for 15 minutes at room temperature. The samples were rinsed twice with a phosphate buffer (Sigma-Aldrich Poland, Poznań, Poland) to remove the fixative. Dehydration in increasing concentrations of ethanol (25%, 50%, 60%, 70%, 80%, 90%, and 100%) was performed for 10 minutes per solution. The ethanol was then rinsed off, and the samples were dried at room temperature. Next, the samples were covered with gold and palladium (60:40; sputter current, 40 mA; sputter time, 50 seconds) using a Quorum machine (Quorum International, Fort Worth, TX) and examined under a Zeiss EVO MA25 scanning electron microscope (SEM) (Carl Zeiss, Oberkochen, Germany). The analyzed strains had to meet all of the following criteria to be considered as “biofilm-forming strains”: adhesion to surface, i.e. positive observation of adhered cells; presence of multilayer structure [at least several dozens of cell layers seen in at least 10 fields of observation]. The survival of preformed biofilm on HA discs: the gentamicin sponge was cut aseptically into 10mmx10mm pieces which were placed over HA discs with preformed biofilm on them. The whole setting was immersed in 2mL of the appropriate liquid medium. The following contact times were applied: 8, 24 and 48h. The whole setting was incubated at 37°C. Afterwards, the sponges were removed and the biofilm was subjected to quantitative culture plating: the discs were rinsed with saline and transferred to 1ml of 0.5% saponine (Sigma-Aldrich, Germany) and subjected to intense vortex shaking for 1min to detach the biofilm. Next, serial dilutions were performed and 100μL of each dilution was cultured on the appropriate agar plate and incubated at 37°C/24h. After 24 hours of incubation, the colonies were counted. The number of surviving cells was compared to the number of cells from the control samples, i.e. to biofilm-forming cells, grown on the HA surface but not incubated in the presence of the gentamicin sponge. The following, additional control setting was used to determine whether the type of surface (HA vs. polystyrene) has an impact on biofilm growth and its sensitivity to gentamicin. In the control setting, the HA discs were analogically introduced to 2mL of 1x10^5^ cfu of the tested pathogen in a 24-well plate and incubated for 24h/37°C. Subsequently, the discs were rinsed with saline to remove non-adhered or loosely bound bacteria. Next, the discs were introduced to gentamicin concentrations twice as high and twice lower than the MIC evaluated as described in sections”Antibiotic Sensitivity Testing” and “Serial Microdilution Method Performed in a 96-well Plate” of this manuscript). Subsequent procedures were performed analogically to the ones described earlier in this section. All the experiments were performed in triplicates.

### Statistical analysis

Calculations were performed using the GraphPad Prism version 7 software. Normality distribution was calculated by means of D’Agostino-Pearson omnibus test. Because all values were non-normally distributed, the Mann-Whitney (rank sum) and Kruskal-Wallis test were applied. The results of statistical analyses were considered significant if they produced p-values < 0.05.

## Results and discussion

**Fig 1:**
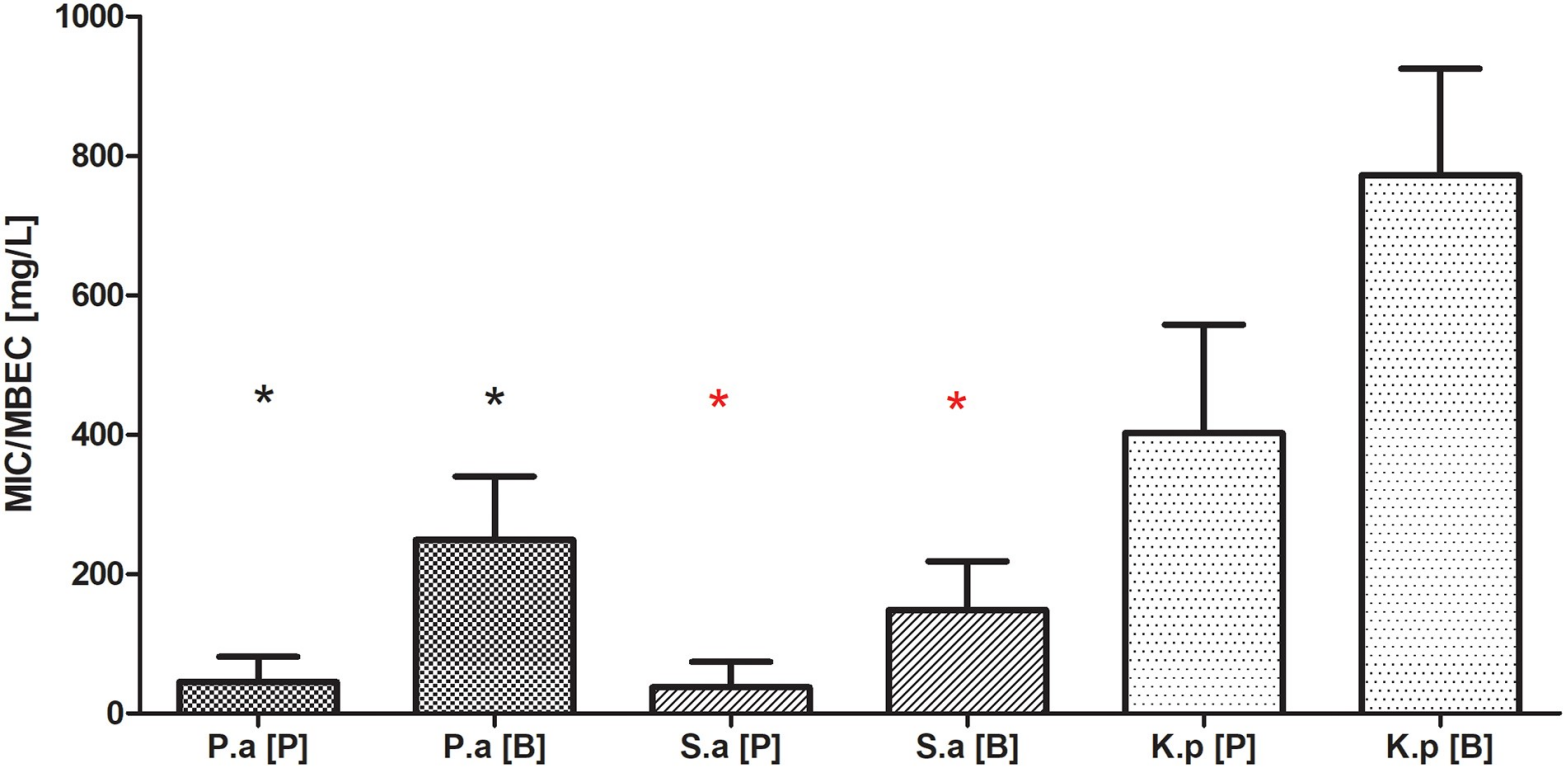
Minimal Inhibitory Concentration [MIC] and Minimal Biofilm Eradication Concentration [MBEC] of gentamicin towards planktonic [P] and biofilm-forming [B] cells of *P*.*aeruginosa* [P.a], *S*.*aureus* [S.a.], *K*.*pneumoniae* [K.p.]. Asterisks show statistical significance [K-W test, p<0.05] between results obtained for **P** vs. **B** of particular microbial species.

Planktonic forms of the analyzed strains displayed diversified sensitivity to gentamicin. 85%, 71% and 41% of *S*.*aureus*, *P*.*aeruginosa* and *K*.*pneumoniae* strains, respectively, showed resistance to gentamicin using the standard E-test method. The results of microdilution method of antibiotic sensitivity estimation (also routinely used in microbiological diagnostics) were fully coherent with the E-test results in the case of *S*.*aureus* and *P*.*aeruginosa* but not for *K*. *pneumoniae* strains. In the case of this pathogen, 76% of the strains were considered resistant to gentamicin according to EUCAST guidelines. Next, the sensitivity of planktonic and biofilm forms (preformed on a polystyrene well of a 96-well plate) toward gentamicin was analyzed. When the *S*.*aureus* and *P*.*aeruginosa* strains were allowed to form biofilm, their tolerance to the antibiotic grew significantly in comparison to their planktonic counterparts (K-W test, p<0.05). In the case of *Klebsiella pneumoniae*, *an* analogical trend was visible, which was however statistically insignificant due to high standard deviations obtained [Fig 1].

Part of the microbes analyzed in this experiment displayed various resistance mechanisms (see Section 2.4.1 of this manuscript), which does not allow clinical application of the entire group of beta-lactam antibiotics. These mechanisms are: methicillin-resistance (MRSA) for *S*.*aureus*; Klebsiella Pneumoniae Carbapenemase (KPC) and New Delhi Metallo-Beta-Lactamase (NDM-1) for *Klebsiella pneumoniae* and Metallo-Beta-Lactamase (MBL) for *P*.*aeruginosa*. With regard to gentamicin, MIC and MBEC of MRSA strains were 2 and 64μg/mL, respectively. Both NDM *K*.*pneumoniae* and MBL+ *P*.*aeruginosa* were resistant to gentamicin not only in biofilm but also in their planktonic forms. MIC and MBEC of KPC+ strains was 4 and 12, respectively, and these values place both forms of the strain in the resistant category according to EUCAST binding rules.

**Fig 2:**
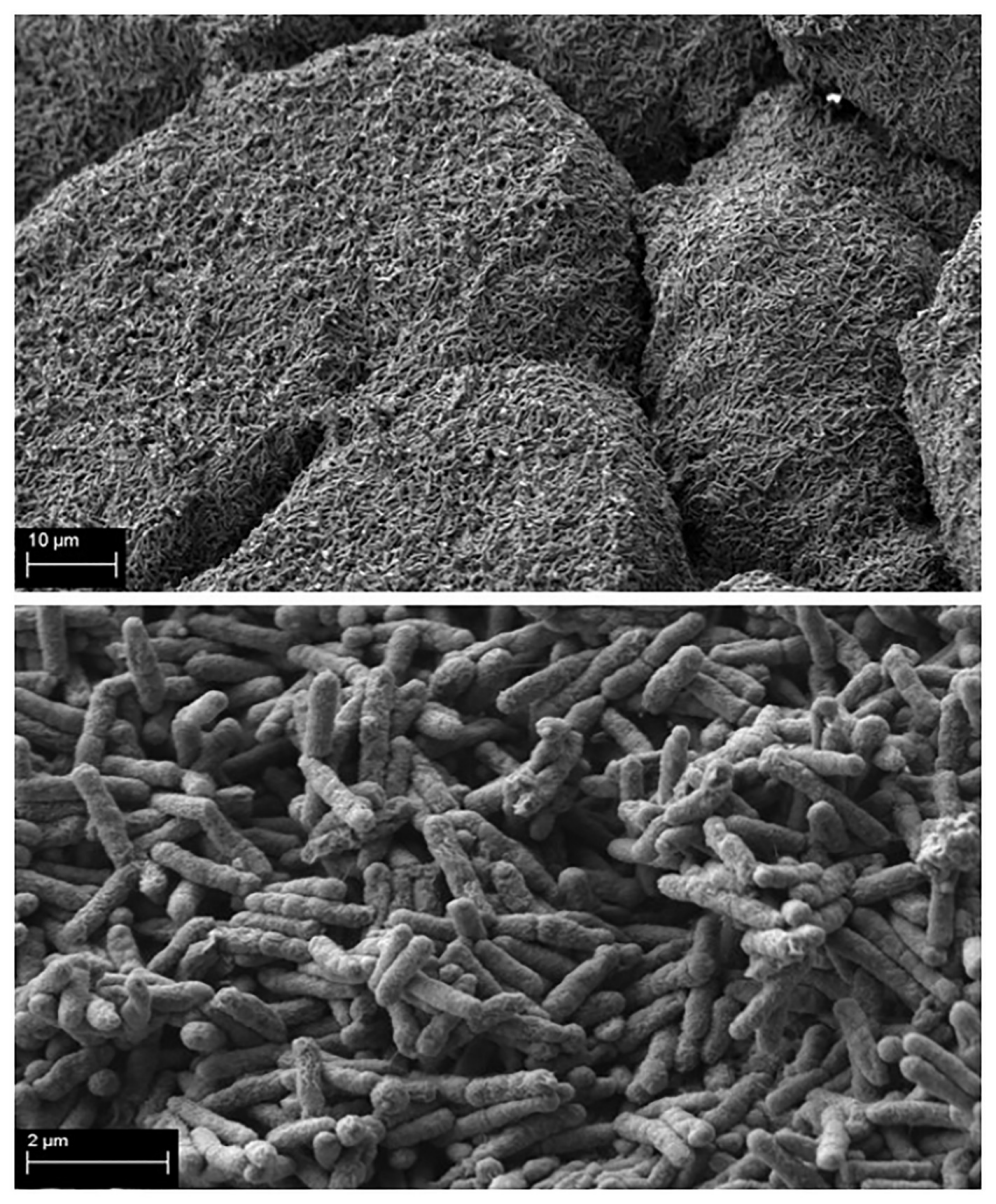
*P*.*aeruginosa* biofilm formed on HA surface. **Upper part of picture**–multilayer form of mature *P*.*aeruginosa* biofilm [Magn.2500x]; **lower part of picture**–*P*.*aeruginosa* biofilm-forming cells [Magn.20000x]. Zeiss Evo Ma SEM.

As a prerequisite for the core experiment, the ability of the tested pathogens to form biofilm on HA discs was investigated by means of Scanning Electron Microscopy. The strains were considered able to form biofilm on HA if they adhered to this surface and if they formed multi-layer structures. All of the strains subjected to the experimental procedures presented later in this manuscript, met these demands. An exemplary picture of biofilm structure formed on HA is presented in Fig 2.

**Fig 3:**
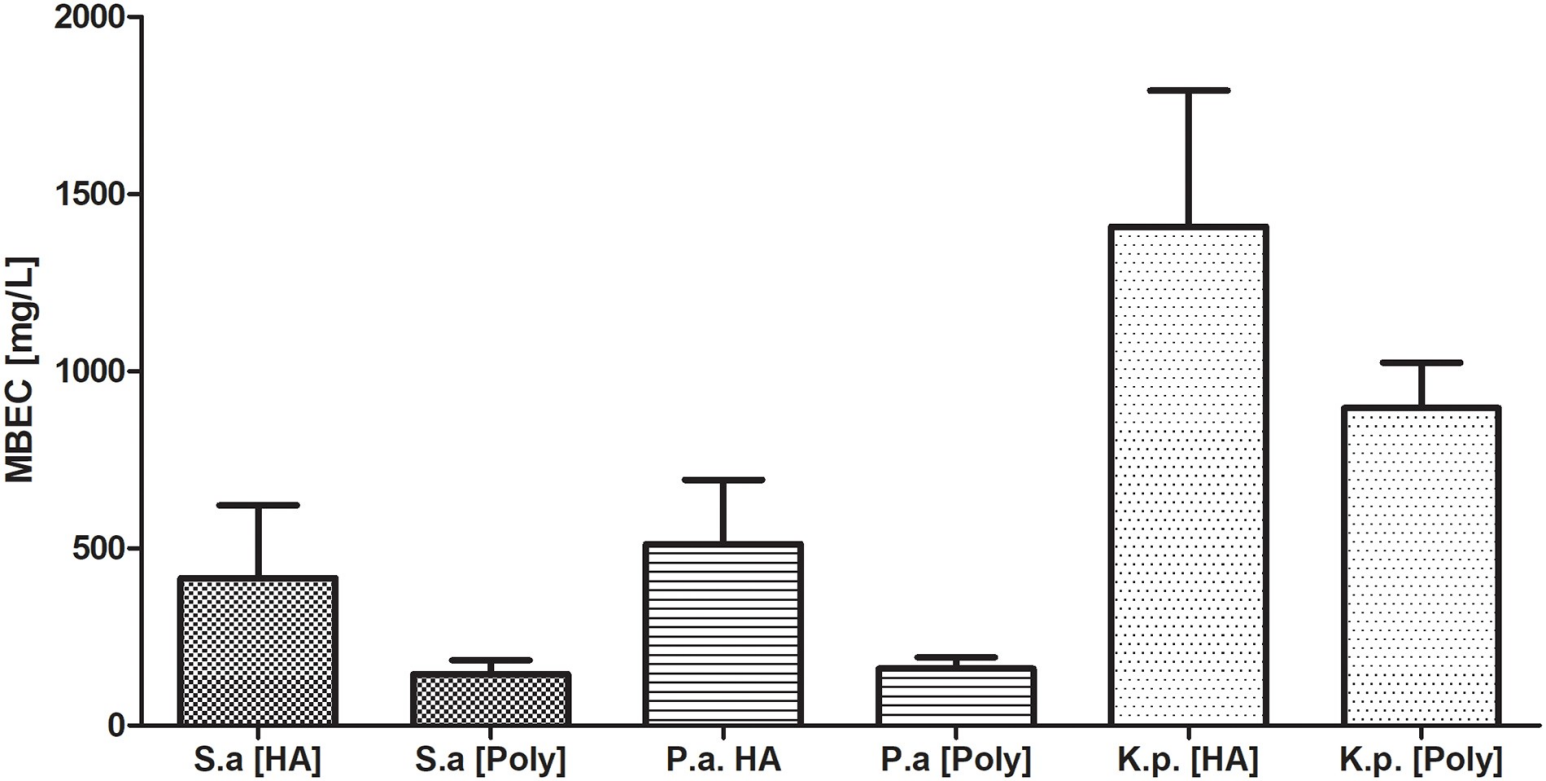
Comparison of Minimal Biofilm Eradication Concentration [MBEC] of gentamicin against biofilm formed on hydroxyapatite [HA] or polystyrene [Poly] surfaces by *P*.*aeruginosa* [P.a], *S*.*aureus* [S.a.], *K*.*pneumoniae* [K.p.] in 24-well plates.

Having proven the ability of the tested microbes to form biofilm, we have performed a comparison of MBEC of gentamicin against the biofilm preformed on HA vs. polystyrene in 24-well plates. The results showed that tolerance to gentamicin of the biofilm formed on these two surfaces was comparable. Although a clear upward trend could be seen in the case of HA-formed biofilm, it was statistically insignificant [K-W test, p<0.05] [Fig 3].

Next, the biofilm was incubated over various time periods (8,24,48h) in the presence of gentamicin sponge. The biofilm of all the strains which were classified under the “sensitive” category by means of the E-test method (see section 2.4.2. of this manuscript), was completely eradicated when incubation with the sponge lasted for 24 and 48h. The same pattern was observed for gentamicin-sensitive *S*.*aureus* and *P*.*aeruginosa* strains after 8 hours of contact time with the gentamicin sponge. The *K*.*pneumoniae* was the exception again, i.e. the biofilm formed by 2 of gentamicin-sensitive strains was not completely eradicated within 8 hours of contact time.

In the case of resistant strains (resistance confirmed by E-test/microdilution method), eradication was not achieved in all the experimental settings applied. The biofilm formed by 3 gentamicin-resistant *P*.*aeruginosa* strains was eradicated only after 48h exposure to the gentamicin sponge. MBEC of these strains, measured using standard methods, was 128-256mg/L. Moreover, one of *P*.*aeruginosa* strains (displaying MBL resistance mechanism) survived exposure to the gentamicin sponge regardless of the time applied. It should be mentioned that the number of biofilm-forming cells was significantly reduced in comparison to the control sample (M-W test, p<0.05) when exposure lasted for 48h. Also two staphylococcal biofilms were able to survive exposure to the gentamicin sponge regardless the time of its application. Another staphylococcal biofilm was eradicated only after 48 hours of exposure. However, a reduction in its biofilm-forming cell number was observed also after 8 and 24 hours. In the case of the latter exposure time, cell number reduction was not complete but statistically significant (M-W test, p<0.05). The number of biofilm forming cells of one staphylococcal strain (for which the E-test method showed growth of cells in the presence of 256mg/L of gentamicin) was comparable to the number of biofilm-forming cells in the control sample regardless the time of exposure to gentamicin.

**Fig 4:**
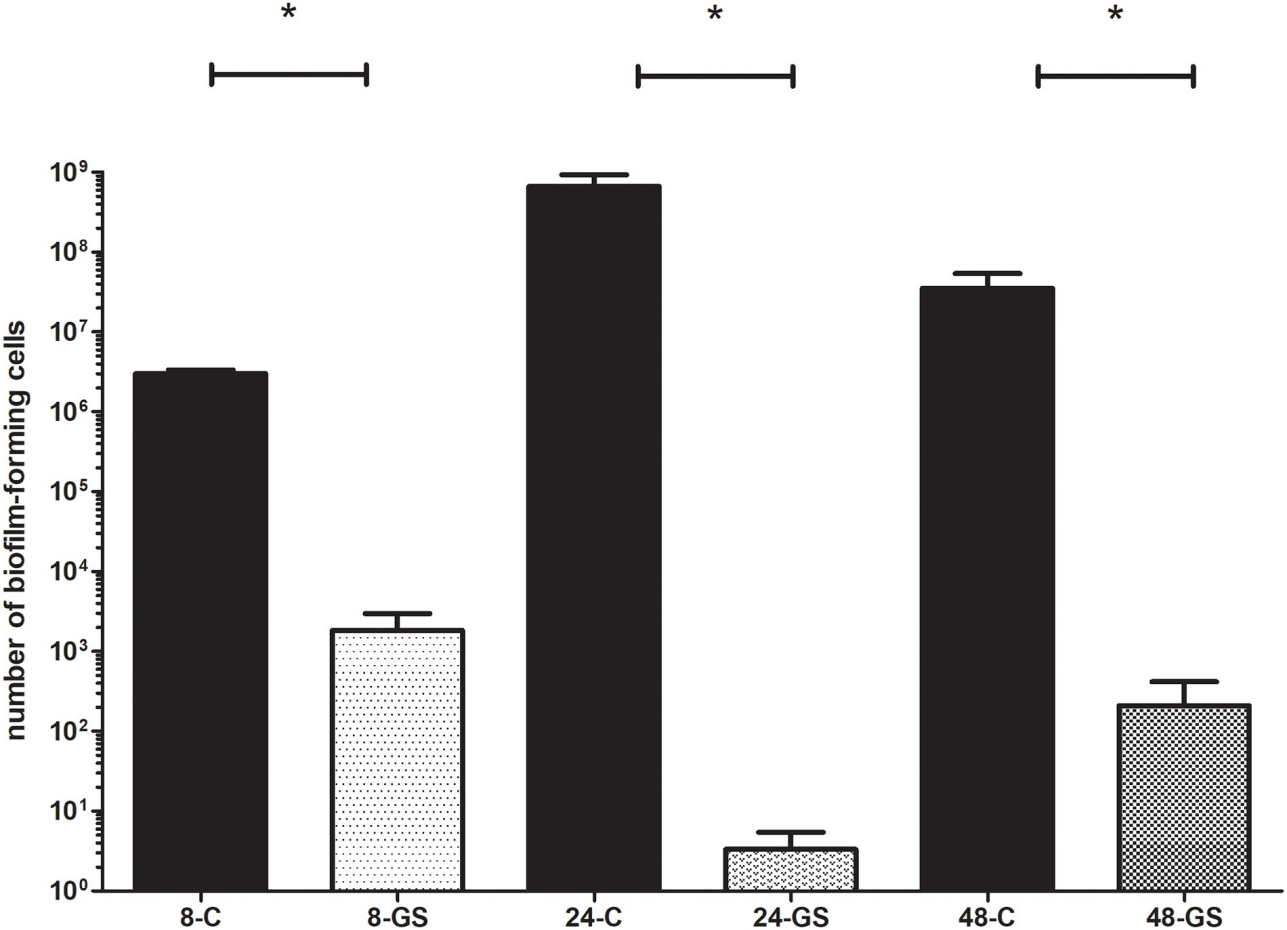
Comparison of the number of *S*.*aureus* biofilm-forming cells after gentamicin sponge application [GS] to the number of biofilm-forming cells of untreated control samples [C]. **8**,**24**,**48** –stand for the hours of incubation of the analyzed sample within the sponge. For the Reader’s convenience, a logarithmic scale was applied here. All the differences between the respective control and analyzed biofilm samples are statistically significant (M-W test, p<0.05).

**Fig 5:**
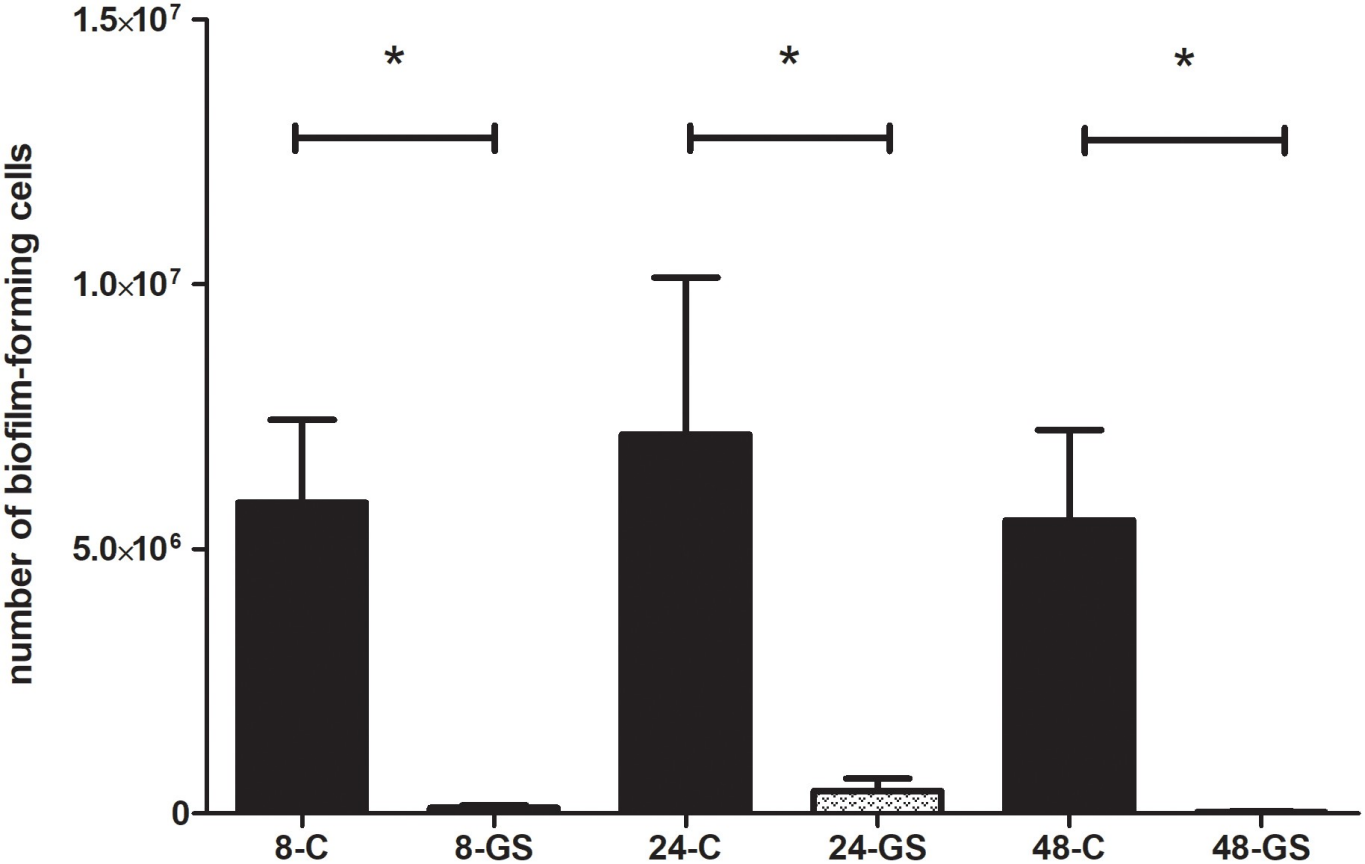
Comparison of the number of *P*.*aeruginosa* biofilm-forming cells after gentamicin sponge application [GS] to the number of biofilm-forming cells of untreated control samples [C]. **8**,**24**,**48** –stand for the hours of incubation of the analyzed sample within the sponge. All the differences between the respective control and analyzed biofilm samples are statistically significant (M-W test, p<0.05).

**Fig 6:**
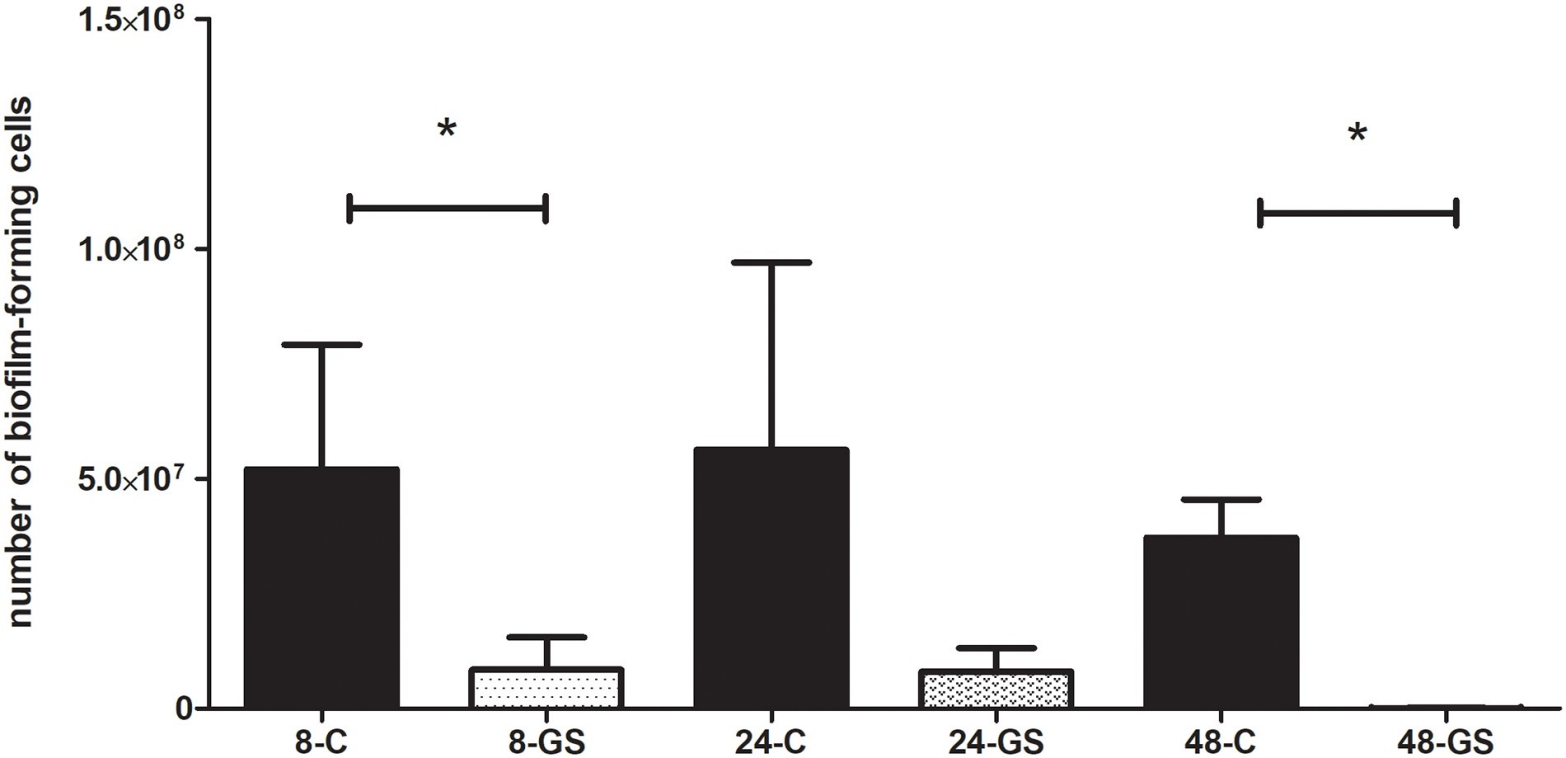
Comparison of the number of *K*.*pneumoniae* biofilm-forming cells after gentamicin sponge application [GS] to the number of biofilm-forming cells of untreated control samples [C]. **8**,**24**,**48** –stand for the hours of incubation of the analyzed sample within the sponge. Statistically significant differences between the respective control and analyzed biofilm samples are marked with an asterisk (M-W test, p<0.05).

It should be emphasized that the biofilm of these of *Klebsiella pneumoniae* strains which were gentamicin-resistant according to the E-test method (with sensitivity ranges from 3 to 12 mg/L) was sensitive to high concentrations of gentamicin released from the sponge carrier. On the other hand, very highly resistant *Klebsiella* strains (MIC>256mg/L) were resistant to gentamicin released from the sponge even when the exposure lasted for 48h. The results of eradication ability of gentamicin released from the sponge against the biofilm of *S*.*aureus*, *P*.*aeruginosa* and *K*.*pneumoniae* are presented in Figs 4, 5 and 6, respectively.

In the present days, microorganisms rapidly acquire resistance to antibiotics; multidrug-resistant strains spread globally and present new mechanisms of resistance (for example VIM, IMP, NDM-1, KPC, OXA-48 carbapenemases, ArmA methylotransferases) [27–31]. The discovery that a majority of infections are caused by biofilms has changed many views in modern microbiology and medicine on the treatment and pathogenesis of infections [4,32]. Therefore, the search for new therapeutic approaches is of paramount importance. The problem also concerns bone inflammation. Because of bone specific architecture, the immune system components and most antibiotics penetrate it poorly, which allows microorganisms to multiply at the infection site and spread within the patient’s body via blood circulatory system [6,33,34]. In turn, antiseptics, whose penetration through biofilm layers is higher, are not approved for internal use due to potential cytotoxicity [35,36,37].

It is also already known that factual MBEC values of antimicrobials are up to a few hundred times higher than MICs measured by means of routinely used *in vitro* methods [38,39]. Due to this disadvantage and the physiological obstacles mentioned above, if to be stopped, bone infection may require surgical removal of the infected bone together with local application of high concentration of antibiotic released from a surgically introduced carrier [5,6,7]. Collagen sponges soaked with gentamicin may be successfully used for such purpose [15].

The results of experiments performed in this study confirm that the microorganisms infecting bones are able to form strong biofilm structures on hydroxyapatite, which is the main inorganic component of bone and constitutes up to 70% of bone’s total mass [40].

The MBEC values of all investigated strains were significantly higher than their MIC values [Fig 1]. The above means that effective therapy of bone infection with the use of gentamicin requires high concentrations of this antibiotic that can only be delivered by local and not by systemic application. With regard to antibiotic sensitivity testing, it seems that various standard evaluation methods fail to estimate the exact tolerance of not only biofilm but also of planktonic forms of microorganisms (or at least what we believe are planktonic forms of microorganisms) to antibiotics. We have observed discrepancies between the results measured by the E-test and the micro-dilution method in the case of *K*.*pneumoniae* strains. They may be due to the fact that in the microdilution method, *Klebsiella* cells form a heterogenic community, where a part of the cells are truly planktonic and others form a so-called floating biofilm (non-adhered, matrix-embedded multicellular structure at the air-liquid interface, also known as pellicles). It may result in a shift of resistance to a level significantly higher than the one obtained in the E-test method and may satisfactorily explain the differences observed between results [41,42,43]. Presently, there is no single recommended clinical routine approach to the estimation of biofilm tolerance to antimicrobials. The above is due to the fact that biofilm formation is a multidimensional, complex phenomenon dependent on many factors, also in a clinical setting. Therefore, it is hard to obtain satisfactory repeatability of measurements, especially with regard to such strong slime-forming species as *Klebsiella pneumoniae*. Also, it is hard to imitate the conditions of bone infection reflecting actual *in vivo* biofilm’s tolerance to antimicrobials. Our experiments have shown that hydroxyapatite [Fig 2], which is the main inorganic component of actual bone, is a surface on which more biofilm is formed than on standard polystyrene. When subjected to gentamicin, HA-formed biofilm displayed a higher tolerance in comparison to the biofilm grown on a polystyrene surface [Fig 3]. It needs to be noted that this trend, however repeatable and clearly observed, was statistically insignificant [Fig 3]. However, in our opinion, these high values of standard deviations were the result of inevitable procedures performed during biofilm cultivation (medium change, rinsing of HA discs with physiological saline to remove non-adhered cells, etc.). It only shows the great need for the development of new models of biofilm testing reflecting actual conditions of its development within the human body.

Other authors report that gentamicin released locally in high concentrations may break through the intrinsic tolerance of bone biofilm [44,45]. In our study we have shown that all sensitive strains (in their planktonic form, according to EUCAST guidelines) were successfully eradicated also when grown as biofilm on HA discs (Figs 4–6). One of the most important messages from our study is that such effect was obtained despite the fact that these biofilm forms were actually gentamicin-resistant if we were to rely on binding EUCAST guidelines only. Moreover, even some highly gentamicin-resistant strains (resistance refers to their planktonic forms according to EUCAST) were also efficiently eradicated by the gentamicin sponge when they grew as biofilm on HA discs. Such an effect was observed for 75% of resistant *P*.*aeruginosa* and *S*.*aureus* strains and for 57% of resistant *K*.*pneumoniae* strains.

The eradication efficacy (after exposure to high gentamicin concentration) seems to be dependent on a strain, exposure time, original MIC value but also on the existence or lack of a resistance mechanism. It has already been proven that KPC+ strains may display sensitivity to gentamicin. However, resistance genes encoding carbapenemase and resistance to gentamicin are not located on the same plasmid [46,47]. The opposite situation occurs in the case of NDM resistance, where the NDM-1 beta-lactamase encoding gene is located on the same plasmid on which the genes encoding resistance to aminoglycosides are also found [48]. When an enzymatic mechanism of aminoglycoside resistance is found in a specific bacterial strain, it is disputable whether very high antibiotic concentrations may break through it [49]. Further research is required to fully elucidate this issue [50,51,52].

Our results stay in line with the data provided by the team of Overstreet et al. [53] concerning high applicability of local delivery of gentamicin from other types of carriers, namely resorbable viscous hydrogels or with a bulk of clinical evidence presented in the review analysis by Koziol et al. [54]. However, the present article is of pilot character, its results confirm the efficacy of high local concentrations of gentamicin released from a sponge carrier against biofilm-forming strains which are classified as resistant according to EUCAST guidelines (especially, if gentamicin’s MIC is close to the EUCAST breakpoint values). Moreover, the use of hydroxyapatite as a surface for biofilm culturing and assessment of gentamicin activity allows to realize that the applied *in vitro* model may have an impact on such essential outcomes as MIC or MBEC values. Although the results of our studies and the data presented by other researchers indicate high applicability of the gentamicin sponge in fighting biofilm infections in bone, one should bear in mind that this type of therapy may be performed only in justified cases and only combined with another, systemically delivered, antibiotics and surgical support.
